# Seventy years of haemophilia care: A personal perspective

**DOI:** 10.1111/vox.13173

**Published:** 2021-06-22

**Authors:** Cees Smit

**Affiliations:** ^1^ Department of epidemiology Leiden University Medical Center (LUMC) Hoofddorp The Netherland

## INTRODUCTION

Having lived for over 70 years with severe haemophilia A, I have taken up the challenge to portray not only my life with haemophilia but also my career in haemophilia and patient advocacy. The result has been a book named, *Surviving Hemophilia, A Road Trip Through the World of Healthcare*. This commentary is an excerpt of the memories, emotions and experiences contained in the book.

## MY EARLY HAEMOPHILIA YEARS

Luckily enough, soon after my birth in 1951, I was quickly diagnosed with a spontaneous form of severe haemophilia A. At the same time, my parents were told that I would not grow old, maybe I would reach the age of 20, maybe 30, as there was no effective treatment. Despite this perspective, my parents never patronized me and tried to give me a carefree youth. After a couple of life‐threatening bleeds, my physician proposed to treat me with growth hormones. I was 6 years old. This turned out to be an experimental treatment with di‐ethyl‐stilbestrol (DES), introduced by the French doctor Raymond A. Turpin in the late 40s. At that time, DES was used as a medication for a variety of female reproductive problems and to inhibit growth in adolescent girls. DES caused dreadful anomalies in women and their offsprings; as for me, DES inhibited my linear growth at 1.45 m. This was one of the drastic experimental treatments for haemophilia in the days when there was no treatment at all. I also took part in a more innocent experiment, the use of peanuts or peanut extract, a therapy developed by H. Bruce Boudreaux who himself had haemophilia. Both therapies did not have any positive outcome.

## A SPRING OF HOPE

The real turnaround moment for haemophilia treatment came when Judith Pool described how to isolate blood plasma concentrates that contained more Factor VIII on a volume basis than fresh plasma. In 1967, I received my first infusion with Factor VIII concentrates. This had an unexpected effect; I became stuffy and could hardly breathe. I thought I was going to die. I was lying in a single room with the door closed; I tried to scream, but that was impossible because of my thick throat. Luckily enough, a nurse entered my room, she saw what was going on, and I guess she reduced the infusion speed. It was an allergic reaction; I never again had one that serious.

## PROPHYLAXIS AND HOME TREATMENT

Gradually, my physician thought that a regular dose of Factor VIII could avoid most of my spontaneous bleeds, and prophylaxis entered my life. It was soon to be followed by transfusions at home that solved my needle fear, one of my early anxieties. I got a normal, regular life and even sports came within reach. In the mid‐70s, the Italian doctors Pier Mannucci and Zaverio Ruggeri introduced a winter sport holiday for haemophilia families in the Italian Dolomites. My Dutch haematologist Jan Wouter Ten Cate thought they were crazy, but they invited him to take part in such a holiday. Jan Wouter Ten Cate asked me instead and since then, I enjoyed cross‐country skiing together with families of the Italian Haemophilia Society for almost 40 years. Winter sports had been unimaginable for me, for my parents and my doctors. The confidence everyone with haemophilia gained from this experience reflects the freedom and emancipation of the haemophilia community in those years.

## THE WINTER OF DESPAIR

All feelings of optimism and freedom disappeared with the viral infections caused by hepatitis C virus (HCV) and human immunodeficiency virus (HIV) in the late 70s and 80s. In the end, these virus infections caused a winter of despair in the international haemophilia community with many thousands of HCV and HIV transmissions. Without an effective treatment for HIV, almost all HIV‐infected people with haemophilia died. Early HCV treatments were little effective; only recently, there is a real therapy to treat HCV. For many people with haemophilia, this treatment came into existence too late. They either died or lived with severe liver damage.

## COMORBIDITY IN OLDER PEOPLE WITH HAEMOPHILIA

**FIGURE 1:**
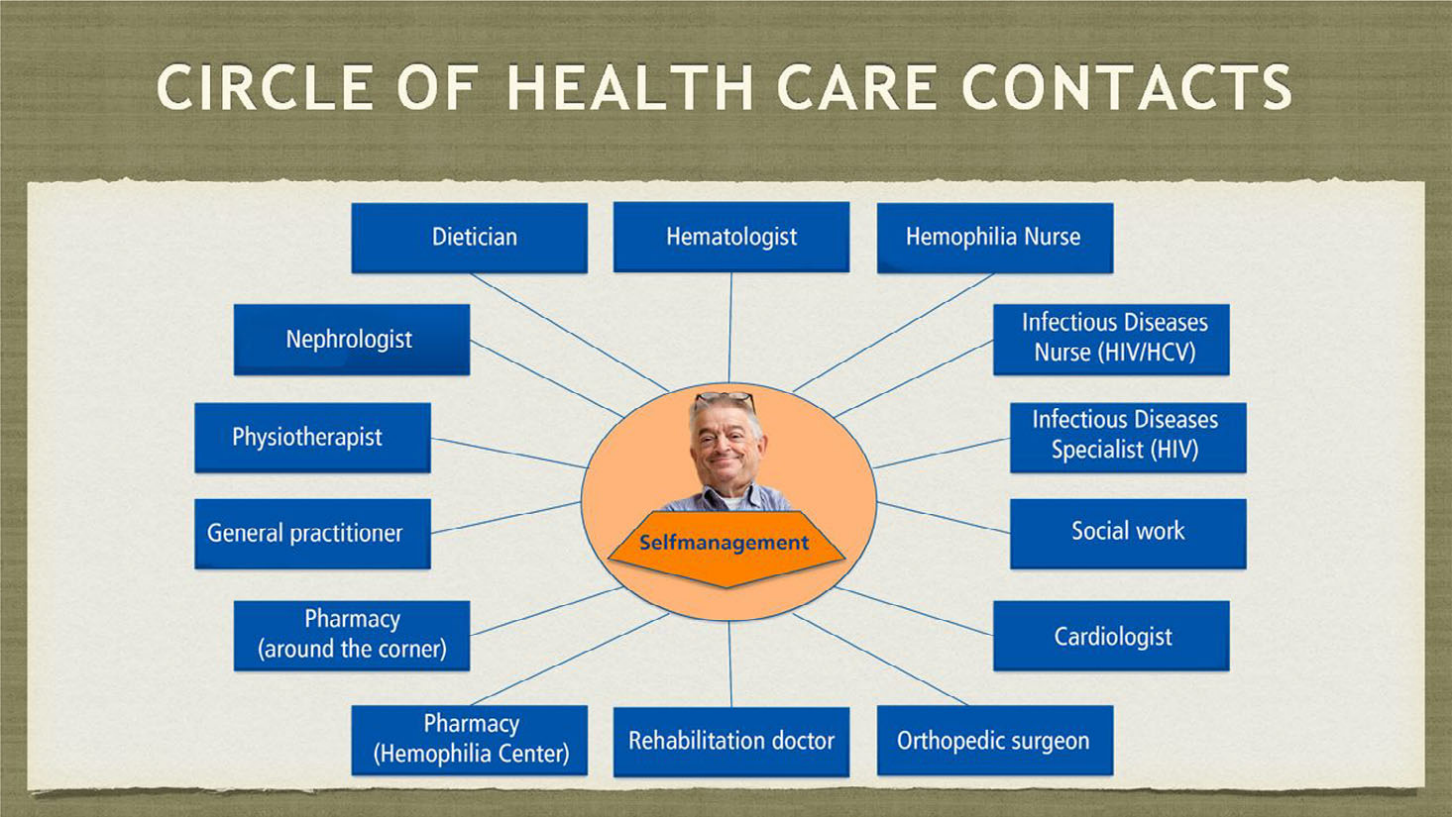
An example of healthcare contacts' network of an ageing man with haemophilia

Untreated bleeding episodes caused joint damage already in my early life, and experimental treatment limited my growth. I was infected with HCV and HIV, and side‐effects of HIV treatment resulted in serious renal complications. I am now a typical example of an ageing man with haemophilia and complex comorbidity. This results in my extensive network of healthcare contacts (Figure 1). The combination of the needle phobia from my childhood, together with comorbidity and polypharmacy issues, creates for me the phenomenon of the ‘fear factor’, the lack of control when you are hospitalized and when you need medical treatment from physicians who are less familiar with haemophilia. An additional fear is related to my ageing process. I experience my health situation as fragile and easily unbalanced.

## COMMERCIALISM OVER VOLUNTARISM

There are some less beautiful sides of haemophilia care and plasma supply. These are the focus of the pharmaceutical industry on profits and the negligence to create a safe blood policy. It is less known that Judith Pool wrote a letter to the Nixon administration about the new US national blood policy. She wrote in 1974: ‘My concern […] is that it in no way requires or even encourages the use of volunteer blood for this purpose but assumes a continuation of the dangerous, expensive, wasteful, and unethical purchase of plasma by pharmaceutical houses to provide such therapeutic material’. Another memorable moment in time can be found in the famous book *Journey*, published by Suzanne and Robert Massie in 1976. They described the battle between Hyland/Travenol (now Takeda) and the American Red Cross over the production of Factor VIII concentrate, a battle that was won by Hyland/Travenol. The Massies consider this as a great loss for the system of solidarity and the voluntary non‐remunerated blood and plasma donation. A third striking and neglected fact in the 70s is that the plasma industry ignored that their own factory workers fell ill with hepatitis [1].

## A FORESEEABLE DISASTER

The tainted blood history shows that somewhere on this road, people not only ignored an orange, but even a red light. Persons in charge within the international haemophilia community focussed on the availability of Factor VIII to meet demands, while people with haemophilia embraced the freedom they got from their medication. No one wanted to lose these acquired rights. In the 1970s, the plasma fractionation industry knew the dangers of hepatitis viruses in the plasma supply. Even some of their employees warned for these contaminations. This makes it unacceptable to speak of a ‘tragic incident’ in haemophilia history. Tainted blood was a foreseeable disaster. Numerous accusations have been made against national governments and are still being made in the Infected Blood Inquiry in the United Kingdom [2]. Until now, the international plasma industry has escaped an investigation into the causes of their plasma procurement process and its inadequacies in the 70s and 80s. People with haemophilia and their families still experience the burden of this tragedy. Many died and, those who survived live with ill health for more than 40 years.

## A NEW SPRING OF HOPE

The voluntary blood and plasma sector never succeeded in getting control over the blood and plasma supply. On the contrary, the haemophilia market is now a booming business with more than 60 companies active in a billion‐dollar market. Plasma concentrates for haemophilia have been replaced by recombinant products, extended half‐life products, factor‐replacement therapies and gene therapies. For people with haemophilia, new therapies cause uncertainties, while existing products have its relatively unease like intravenous access. Unfortunately, people in three quarters of the world still have no access to haemophilia therapy because of the price of the haemophilia products and its relative scarcity.

## GLOBALISM OVER SELF‐RELIANCE

For other plasma products, especially immunoglobulins, the world is dependent on the large paid plasma donor pool in the United States. This plasma pool is no longer owned by US‐based plasma collection centres but by large international pharma companies. National blood transfusion services gradually lost their share on this market. European countries permitted the selling of their plasma factories, BPL UK and Biotest in Germany, to China's Creat. It shows that globalism has become a dominant factor in the blood and plasma industry and supersedes nation's historical principle of self‐sufficiency. At present, dealing with COVID‐19, we experience the impact of globalism on our health care. It is a situation that was to be foreseen when we look at the history of haemophilia treatment. History does not repeat itself, but it often rhymes.

## COROLLARY

I am glad I have survived these 70 years living with haemophilia, and I must acknowledge that I could have never come this far without support. A large group of professionals, doctors, nurses, my friends, my parents, my family, my partner and her children have always been at my side. I am also grateful to the many donors in the Netherlands who voluntarily donate their blood plasma.

## CONFLICT OF INTEREST

The authors declare no conflicts of interest.

## FUNDING INFORMATION

None.
